# Chlamydial and Gonococcal Genital Infections: A Narrative Review

**DOI:** 10.3390/jpm13071170

**Published:** 2023-07-21

**Authors:** Rafaela Rodrigues, Pedro Vieira-Baptista, Carlos Catalão, Maria José Borrego, Carlos Sousa, Nuno Vale

**Affiliations:** 1 OncoPharma Research Group, Center for Health Technology and Services Research (CINTESIS), Rua Doutor Plácido da Costa, 4200-450 Porto, Portugal; rafaela24sofia@gmail.com; 2 CINTESIS@RISE, Faculty of Medicine, University of Porto, Alameda Professor Hernâni Monteiro, 4200-319 Porto, Portugal; 3 Molecular Diagnostics Laboratory, Unilabs Portugal, Centro Empresarial Lionesa Porto, Rua Lionesa, 4465-671 Leça do Balio, Portugal; carlos.sousa@unilabs.com; 4 Department of Gynecology-Obstetrics and Pediatrics, Faculdade de Medicina da Universidade do Porto, Alameda Professor Hernâni Monteiro, 4200-319 Porto, Portugal; pedrovieirabaptista@gmail.com; 5 Lower Genital Tract Unit, Centro Hospitalar de São João, Alameda Professor Hernâni Monteiro, 4200-319 Porto, Portugal; 6 Roche Sistemas de Diagnósticos, Estrada Nacional, 2720-413 Amadora, Portugal; carlos.catalao@roche.com; 7 Laboratório Nacional de Referência das Infeções Sexualmente Transmissíveis, Instituto Nacional de Saúde Dr. Ricardo Jorge, Avenida Padre Cruz, 1649-016 Lisboa, Portugal; m.jose.borrego@insa.min-saude.pt; 8 Department of Community Medicine, Health Information and Decision (MEDCIDS), Faculty of Medicine, University of Porto, Rua Doutor Plácido da Costa, 4200-450 Porto, Portugal

**Keywords:** sexually transmitted infections, *Chlamydia trachomatis*, *Neisseria gonorrhoeae*, infertility, tumorigenesis, prevention, screening, diagnostic, treatment

## Abstract

Sexually transmitted infections (STIs) constitute one of the leading causes of disease burden worldwide, leading to considerable morbidity, mortality, health expenditures, and stigma. Of note are the most common bacterial STIs, chlamydial and gonococcal infections, whose etiological agents are *Chlamydia trachomatis* (CT) and *Neisseria gonorrhoeae* (NG), respectively. Despite being usually asymptomatic, in some cases these infections can be associated with long-term severe complications, such as pelvic inflammatory disease, chronic pelvic pain, infertility, ectopic pregnancy, and increased risk of other STIs acquisition. As the symptoms, when present, are usually similar in both infections, and in most of the cases these infections co-occur, the dual-test strategy, searching for both pathogens, should be preferred. In line with this, herein we focus on the main aspects of CT and NG infections, the clinical symptoms as well as the appropriate state-of-the-art diagnostic tests and treatment. Cost-effective strategies for controlling CT and NG infections worldwide are addressed. The treatment for both infections is based on antibiotics. However, the continuing global rise in the incidence of these infections, concomitantly with the increased risk of antibiotics resistance, leads to difficulties in their control, particularly in the case of NG infections. We also discuss the potential mechanism of tumorigenesis related to CT infections. The molecular bases of CT and NG infections are addressed, as they should provide clues for control or eradication, through the development of new drugs and/or effective vaccines against these pathogens.

## 1. Introduction

Globally, sexually transmitted infections (STIs) are a public health concern that is still challenging, especially among adolescents and young adults [1,2,3]. Furthermore, according to the World Health Organization (WHO), during the pandemic of COVID-19, efforts were directed toward SARS-CoV-2 infections, leading to STIs being in oblivion, with a consequent increase in their incidence [4].

STIs are associated with high morbidity, especially in the psychological, sexual, and reproductive health domains in both women and men. Therefore, health professionals treating patients with STIs must consider that these diseases could affect the quality of life of the infected persons. Thus, they must consider the mental dimension, and treat the infections and the possible psychological consequences, as reported in previous studies [5,6]. Also, the impact on newborn infants of infected individuals due to vertical transmission cannot be neglected [7]. Women are significantly more affected than men, which, in part, is explained by anatomy, which makes women more exposed and susceptible to these infections [8].

Moreover, it is important to refer to the fact that STIs have high associated health expenditure [9,10,11]. Indeed, the best current approach to handling the problem is through well-organized screening and surveillance programs, to diagnose and treat the infected people in a timely and adequate way; consequently, this will allow for the control and breaking of the transmission chain [12]. Nevertheless, this is also costly, so the European Center for Disease Prevention and Control highlights that it is necessary to estimate STI prevalence and incidence better. Accordingly, each country must design and adopt prevention measures adapted to their reality [13,14,15,16]. In some regions, particularly Africa and Latin America, it remains challenging to control STIs. While not exclusively due to limited access to diagnostic instruments, this factor significantly contributes to the high incidence of these infections in those areas [1,8].

STIs are frequently asymptomatic; notwithstanding, they can cause a diversity of infirmities, including acute and chronic severe problems [13]. In particular, the most serious associated complications are reproductive organ ones, with symptoms reported by women and men that can comprise mainly genital, extragenital, or disseminated symptomatology [17]. Indeed, the majority of STI cases are asymptomatic, such as chlamydial and gonococcal infections, leading to infection persistence, increasing risk of transmission, and, importantly, in some cases possibly leading to the development of associated complications, which, in the worst-case scenario, could be irreversible and potentially fatal; these include infertility, ectopic pregnancy, scarring, chronic pain, neonatal death, congenital abnormalities, sexual dysfunction, and cancer, depending on the pathogen(s) that cause the infection(s) [17,18]. Although STIs can be caused by several types of pathogens (viruses, bacteria, and parasites), herein we will focus on the two most common bacterial ones: the *Chlamydia trachomatis* (CT) and *Neisseria gonorrhoeae* (NG) infections [19]. It is also important to highlight the fact that sometimes natural clearance of these infections could occur, and, despite being curable STIs, they could also frequently be associated with serious public health concerns, including NG antibiotic resistance [13]. Interestingly, while some authors defend the case that CT and NG co-infections occur randomly, it has been proved using mathematical models that the occurrence is indeed much higher than expected in a random model. However, the underlying mechanisms are still unknown [20,21,22]. 

According to the WHO, in 2020 there were 129 million new CT infections worldwide and 82 million new NG cases NG [23]. The Center for Disease Control and Prevention (CDC) estimates that in 2018 in the USA, 4 million new CT infections occurred, translating in an estimated medical cost of USD 691 million. Regarding NG infections, the CDC estimated that 1.6 million new cases occurred in the US, associated with a medical cost of around USD 270 million [11].

These infections are most frequent in females, peaking at 15–24 years [24]. Notwithstanding, Kaufman and others assert that there may be a shift in the age group of the incidence peak—specifically to the 25–30-years interval. However, it must be kept in mind that this particular study had external validity issues; therefore, further investigation is needed before these results can be assumed [25].

STIs are associated with 2.3 million deaths and 1.2 million cancer cases yearly. Accordingly, all countries must make an effort and adopt strategies to reduce their incidence, as highlighted in the WHO guide for the health sector *Global health sector strategies on sexually transmitted infections for the period 2022–2030* [26].

Our review addresses the state of the art in CT and NG urogenital infections, to identify the knowledge gaps and understand the impact of the implemented infection control strategies that could be improved in the following years.

## 2. Pathogenesis of CT and NG Infections

CT and NG can infect different anatomical regions, namely, the urogenital, anorectal, and oropharyngeal tract [19]. Notwithstanding, the extensive studies on these infections have focused primarily on the urogenital tract, because it is the most common site of infection and transmission. Also, reproductive organ infections have more direct public health implications, such as pelvic inflammatory disease (PID), infertility, increased risk of human immunodeficiency virus (HIV) transmission, and other equally serious potential sequelae [22,27,28]. Nevertheless, it is crucial to remember that extragenital infections are also associated with increased risk of HIV transmission, and that in specific groups these extragenital infections are even more relevant and likely to occur, as we will discuss further [18,19].

### 2.1. Clinical Manifestation

**Figure 1:**
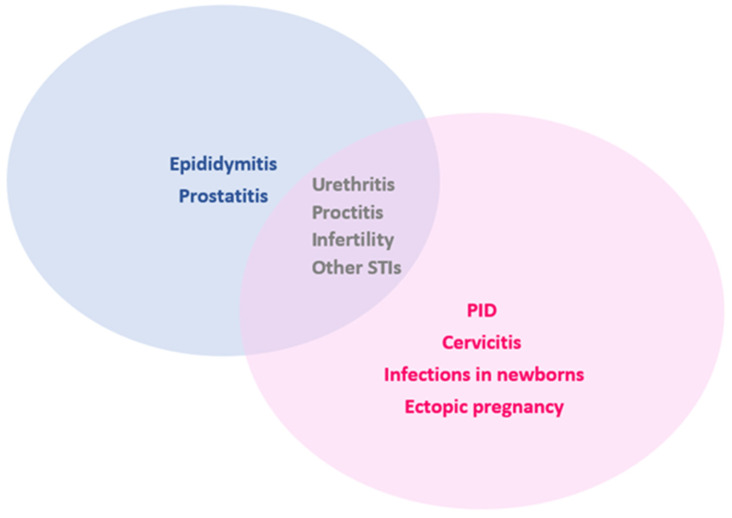
Common clinical conditions associated with chlamydial and gonococcal genital infections according to gender. PID—pelvic inflammatory disease; STI—sexually transmitted infection.

It is well known that chlamydial and gonococcal infections are mostly asymptomatic, meaning that the diagnosis of the cases occurs mainly during screening [29,30]. If symptomatic, the presentation is usually non-specific, and a laboratory diagnosis is still warranted. The empirical clinical diagnostic procedure is insufficient, and must be guided by molecular methods, in detail by the Nucleic Acid Amplification Tests (NAATs), which mitigate the risk for false negative tests, detecting the exact bacterium and correctly guiding the treatment [31]. In detail, some of the main clinical outcomes of CT and NG genital infections, depending on the anatomical site and gender, are depicted in Figure 1.

Indeed, infertility is one of the most severe outcomes of these infections, and can affect both sexes (Figure 1). CT infection and associated infertilitywere recently revisited in detail in our previous work [12]. Interestingly, women’s capacity to become pregnant was studied in a clinical trial, in which investigators evaluated pregancy incidence and time to become pregant in CT-infected woman vs. non-infected woman, concluding that CT infection negatively impacts these parameters [32]. Moreover a causal relation between CT infection and ectopic pregnancy was established by Ahmad et al., using in vivo and in vitro models; this group also explained the associated mechanisms behind this [33]. Additionally, Pant et al. investigated the role of matrix metalloproteinase (MMP) expression in the fallopian tubes, which can also play a role in ectopic pregnancy [34].

Furthermore, regarding NG infections, in a recently published meta-analysis, the authors compared the number of NG-infected individuals among infertile populations. When comparing this number with the general population, they found that it was more than two-fold higher in the former, suggesting that this pathogen possibly plays a crucial role in infertility [35]. Finally, it is essential to highlight the fact that in pregnant women these infections are associated with obstetrical and neonatal complications, including conjunctivitis or pneumonia in neonates [36].

### 2.2. Potential Risk Factors for Tumorigenesis

It is well established that some pathogens, including viruses, parasites, and bacteria can trigger tumorigenesis [37]. In line with this, there are some reports concerning the potential role of CT in the induction of this oncogenic process, through some molecular mechanisms [12]. Indeed, the biological plausibility of this association arises from the fact that CT can induce chronic inflammation, cell proliferation through the MEK/ERK signaling pathway, apoptosis inhibition, cell proliferation, and DNA damage mediated by ROS production, and reduce the immune system response [38]. Previous studies reported a possible association with cervical and ovarian cancer. Thus, to further investigate these hypotheses, two different meta-analyses and a systematic review were conducted [38,39,40].

Interestingly, a systematic review with meta-analysis published in 2022 by Hosseininasab-nodoushan et al., corroborates the hypothesis that there is an association between CT infection and ovarian cancer risk (odds ratio [OR]: 1.344; 95% CI: 1.19–1.5). However, the authors acknowledged several limitations: the primary studies considered in the analysis were case–control studies which did not allow for the determination of causality, and there was considerable heterogeneity and possible publication biases [39]. Nevertheless, their results have a clinical significance that must be further investigated. In addition, previous reports have pointed to a potential role for PID in the subsequent development of ovarian cancer. Additional studies are needed to fill the gaps in this topic, specifically, to understand whether non-chlamydial or chlamydial-driven PID may be differential players in ovarian cancer risk [41,42].

Haiyan et al., performed a meta-analysis to clarify the potential association between CT infection and cervical cancer risk. In their study, they not only identified a statistically significant association between CT infection and cervical cancer, but also demonstrated that HPV co-infection with CT is associated with higher risk of cervical cancer [40]. Later, Karim et al. conducted a systematic review in which they summarized all the information on this topic. They concluded that CT can be a risk factor for cervical cancer development, highlighting the fact that this bacterium makes HPV infection more successful, and contributes to molecular processes triggering carcinogenesis. Finally, they also elucidated how CT and HPV infections share some risk factors; therefore, they assert that the individuals who tested positive for one of these pathogens could have higher probability of having the other [38].

Importantly, to the best our knowledge, there are no previous reports regarding the association between CT infection and other tumors. We could not find evidence regarding an association between NG infection and the risk of neoplasia in the literature.

### 2.3. Infection Molecular Pathways and Host Immune Response

**Figure 2:**
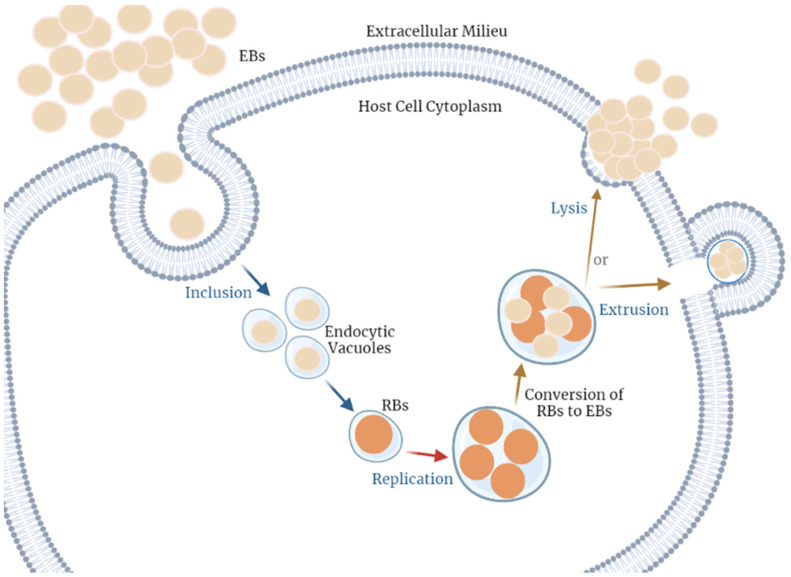
*Chlamydia trachomatis* cell cycle of infection. This pathogen alternates between two distinct forms. The infectious form, named the elementary body (EB), when in contact with a host cell, can reach the cell cytoplasm by adhesion and internalization into a vacuole. Herein, EBs are converted into the alternative non-infectious form, the reticulate body (RB). These can go through the replication process, using the host’s resources, and using the cell’s energy and nutrients; when they reach a critical volume, the RBs transform into the previous form, the EBs. Finally, there are two possible mechanisms for the extracellular EB release, (1) lysis of the host cell or (2) extrusion. This cycle occurs repeatedly in the adjacent cells [12]. Figure created using BioRender.

Understanding the molecular mechanisms of the pathogens’ growth and proliferation is vital for targeting these pathways in infection control. Thus, herein we will explore the life cycle of NG and CT infections. Both bacteria invade the epithelial mucosa, which triggers an immune response by the host cells [43]. It must be noted that, although CT is an obligate intracellular pathogen, which means it depends on the host cell for its replication, NG is a facultative pathogen [44] (Figure 2).

Indeed, the worst consequences of a CT infection begin with chronic inflammation that is not resolved, triggering an excess recruitment of immune cells, especially neutrophils, concomitantly with an overproduction of growth factors, cytokines, and chemokines. This pro-inflammatory milieu and the pathogen persistence could cause severe tissue damage and wound healing delays. Moreover, new insights have been recently made regarding the underlying mechanism of CT pathogenesis, where it was found that the prolonged strong signaling which was mediated by the leukemia inhibitory factor (LIF)/LIF receptor (LIFR) and triggered by a CT infection is a key pathway involved in the more harmful sequelae, namely, infertility, ectopic pregnancy, and cancer [45].

**Figure 3:**
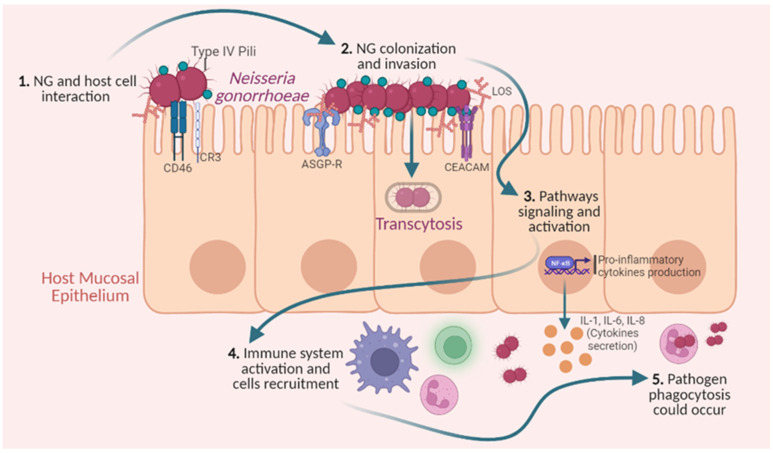
*Neisseria gonorrhoeae* (NG) infection. Briefly, NG infection starts with the host cell interaction, establishing contact through some host cell receptors (CD46 and CR3) and type IV pili communication. After cell adhesion, this bacterium starts its replication and invasion processes, via transcytosis. Concomitantly, NG releases some cellular fragments, such as peptidoglycans and lipo-oligosaccharides (LOS), which, in contact with some cell surface molecules, namely, asialoglycoprotein receptor (ASGP-R) and carcinoembryonic antigen-related cell adhesion molecule family (CEACAM), can activate some signaling pathways (such as NF-kB pathway), triggering processes such as pro-inflammatory cytokine and chemokine production (including IL-1, IL-6, IL-8). In addition, this pro-inflammatory gradient of molecules drives the immune cell recruitment to the local, mainly dendritic, cells, macrophages, and neutrophils. Although these immune cells’ role is to trigger pathogen destruction, mostly through phagocytosis by neutrophils, up to the infection clearance, NG can frequently survive, and the infection can persist. Figure created using BioRender.

NG cannot survive outside the human host. Its infection cycle, represented in Figure 3, usually initiates when it comes into contact with the bacterium adhesins expressed on the host’s mucosal epithelial cells’ surface.

In the first step, there is contact between the type IV pili of the bacterium with the CD46 and CR3 receptors of the cell, which enable ulterior interactions with other cell surface molecules. Also, other bacterium structures, such as colony opacity-associated proteins (OPA) and lipooligosaccharide (LOS), may link to the host cells’ asialoglycoprotein receptor (ASGP-R) and the carcinoembryonic antigen-related cell adhesion molecule family (CEACAM), enabling NG replication to emerge in the epithelium and allowing the subsequent transcytosis and invasion processes [46,47]. To the best of our knowledge, the invasion process depends on the infection site; that is, the bacterium–host-cell communication differs between the cervix and the urothelial tract, involving different molecules as key players, as reported by Green et al. [46].

Concomitantly, these molecular interactions trigger an inflammatory immune response, through the NF-κΒ molecular pathway activation and pro-inflammatory cytokine production. This pro-inflammatory gradient triggers the recruitment of immune cells to the infection site, including macrophages, leukocytes, and neutrophils [47]. These immune cells interact with the pathogens, and are responsible for their phagocytosis and elimination; however, NG has mechanisms of immune escape and suppression, specifically by causing the inhibition of dendritic cells, B cells, and T cells, as further detailed by McSheffrey and Gray-Owen [48,49].

## 3. Gold Standard Diagnostic Method

Chlamydial and gonococcal infections are diagnosed by detecting these bacteria in urogenital, rectal, oropharyngeal, or ocular secretions [50]. According to European and CDC guidelines, the detection of CT and NG should be performed using nucleic acid amplification tests (NAATs), which are based on the amplification of specific bacteria nucleic acid target sequences, mostly used due to their higher sensitivity [19,50,51,52]. Notwithstanding, it must be highlighted that this strategy is not 100% specific and sensitive, and the specimen origin influences the analysis power of the diagnostic test. In detail, European guidelines strongly recommend the use of first-void urine (up to 20 mL sampled >1 h after previous micturition) or cervicovaginal swabs (collected by a health-care worker or self-collected) for the diagnosis of urogenital infections in men and women, respectively.

Of note, the type of specimen chosen will also depend on the available NAATs selected, because they differ in the target nucleic acid sequence and in the amplification technique [51,52]. Herein, we will describe the main FDA-approved commercial kits available to test for both urogenital infections, or more, simultaneously, which is in line with our position regarding the best strategy for a comprehensive screening [53] (Table 1). For example, one of the most complete tests, due to the validation of its use with several specimen types, is Roche COBAS CT/NG test, which is performed through a real-time PCR technique, with dual targets (CT and NG) and internal control in order to check the sample adequacy and detect the presence of PCR inhibitors. In line with this, the VENUS Trial (Vaginal, Endocervical and Urine Screening Trial for CT/NG), in which this test was used, showed that higher sensitivity and specificity were achieved in vaginal swab and male urine samples. Nevertheless, with other samples, the performance was also excellent (sensitivity > 94%), highlighting the great advantage and superiority of the CT/NG molecular testing [54]. Thus, despite being the most reliable technique, it is important to keep in mind that NAATs are associated with some false negative results, and the smaller the sensitivity of the test chosen, the more missing infected individuals there will be [55]. Also, some CT variants were considered hard to detect using some NAATs, leading to false negatives. Thus, acting upon clinical suspicion, a different NAAT test, comprehending a different genomic target, should be used [56,57,58,59].

It is worth noting that it is still necessary and important to use the culture method in cases of resistance, specifically, to test the phenotype or genotype involved in antibiotic resistance mechanisms, also allowing the possible detection of new pathogen strains with mutations that cannot be detected in NAATs [66].

Lymphogranuloma venereum (LGV), according to the CDC guidelines, requires a more prolonged treatment. Thus, in order not to miss an LGV diagnosis, and to allow physicians to implement an adequate therapy, the routine use of laboratory tests that distinguish LGV strains should be mandatory, namely in anorectal CT+ samples. Additionally, regularly performing *ompA*-genotyping of CT strains would allow for the evaluation of the association between the *ompA*-genotype and the severity of the CT infection [67,68]. There are nowadays NAATs commercial tests directed at genomic targets that differentiate LGV strains from common D-K. These tests, together with the analysis of the *ompA* gene, should be able to detect new CT variants [69]. This is of utmost importance when LGV strains may induce more invasive infections involving the lymphatic system. 

Regarding the test of cure, which is the repetition of the test after completion of the treatment, the strategy must be well designed to obtain the correct result, to make the clinical decision to try new therapy (in the case of a positive test), or to document pathogen eradication. Following the guidelines, health organizations generally recommend not testing earlier than approximately 4 weeks after the treatment completion, to avoid false positive results due to the detection of the genetic material of dead pathogens [16,23].

Notably, some studies have shown that when the diagnosis is focused only on reproductive tract specimens, there is a probability of a failing CT or NG diagnosis. Cost-effectiveness studies, for example, such as the one published by Eckman and colleagues, should be performed to understand to what point the extra-urogenital screening strategy would be useful and more appropriate than the urogenital tract screening, only in particular groups or in the general population [65,70,71,72]. In line with this, it could be necessary to adapt CT and NG screening strategies, collecting the appropriate biological samples, to some populations, particularly, men who have sex with men (MSM), who have a higher risk of extragenital infections, rather than urogenital ones [73].

Recently, there have been studies supporting the thesis that the screening strategies for CT and NG infections will only be effective if reformulated for adaption to the 21st century and the specific population being screened, considering the sexual behavior and gender identity of each individual. Accordingly, depending on the risk behavior, different anatomic sites may have to be screened [74,75]. Curiously, there is evidence suggesting that pooled testing for CT and NG detection (pooled rectal, pharyngeal, and urogenital samples), a strategy that tries to minimize the costs of multiple-site screening, is the most cost-effective way to diagnose all chlamydial and gonococcal infections [76]. In line with this, further studies should be pursued to corroborate this hypothesis to assist with the new directions in screening methods for these infections.

## 4. Challenges and Opportunities of the Current Therapeutic Weapons

Gonococcal and chlamydial infections are exclusively treated with antibiotics, despite some authors suggesting possible natural compounds as potential alternatives to be targeted in future studies [22,77,78]. Briefly, in the case of adolescents and adults, the most common subsets of CT and NG infected patients, CDC guidelines for CT treatment recommends doxycycline 100 mg orally, twice daily for 7 days. The alternative regimens are based on azithromycin 1 g orally, in a single dose, or levofloxacin 500 mg orally, once a day for 7 days [79,80]. In the case of NG infections, the CDC guidelines recommends following the regimen of a single 500 mg intramuscular dose of ceftriaxone. In addition, if the co-infection with CT was not discarded, the treatment should include 100 mg orally 2 times a day for 7 days [81]. Of note, the treatment of urogenital infections in pregnant women, neonates and children, as well as of extra-genital infections, is an exception that is beyond the scope of this paper [79,81].

Antibiotic resistance is a challenge that is a common barrier to overcome during the treatment of bacterial infections [82]. Interestingly, it must be highlighted that *Chlamydia suis*, a pathogen for pigs that can also affect humans through zoonotic transmission, is also treated preferentially with azithromycin, a tetracycline drug, but *C. suis* is the only species of *Chlamydia* (genus) for which there has been found a tetracycline-resistant stable phenotype [83,84,85]. This is an important topic to be pursued in future research in CT antibiotic resistance, as explained by Donati et al., as genetic recombination between both species can occur through horizontal gene transfer, putatively turning CT tetracycline resistant [86].

Considering CT and NG infections, the latter poses major concerns regarding this topic, as reported by the WHO [87]. In addition, through whole genome sequencing of NG, investigators concluded that the pathogen had been shaped by the molecules used to treat the infections, giving rise to two main genomic lineages: a multidrug-resistant and a multidrug-susceptible one [88]. Notably, the European Centre for Disease Prevention and Control (ECDC) has developed a program (European Gonococcal Antimicrobial Susceptibility Program) that monitors the drug susceptibilities of NG, namely to ceftriaxone, cefixime, azithromycin, ciprofloxacin, spectinomycin and gentamicin. There is an ongoing similar program in the United States: the Gonococcal Isolate Surveillance Project, funded by the CDC [89,90,91]. Indeed, the most frequent mechanisms of resistance of this pathogen are mediated by mutation in the *penA* gene, which encodes PBP2 transpeptidase (against the beta-lactam antibiotics) and *mtrR* and *mtrCDE* gene mutation, involved in multidrug efflux transport (leading to azithromycin resistance) [92].

NG susceptibility to some microenvironmental changes, such as temperature, oxygen, desiccation, and many fatty acids, can be a window of opportunity for treatment, which deserves further investigation [52]. Furthermore, because NG developed resistance to all approved drugs, future strategies may encompass a better and more expedient diagnosis of the infection to treat these case in an effective and timely way, re-test after treatment, and test sex partners. Moreover, some authors assert that alternative strategies may include, for example, performing a molecular prediction of resistance, developing new drugs, and repurposing old ones [93].

Current treatment options and opportunities for CT infections have been thoroughly discussed elsewhere [22]. In summary, CT antibiotic resistance, as in most bacterial infections, occurs due to an inappropriate use of the drugs. While some gene mutations give protection or resistance to specific drugs, frequently the treatment regimen chosen does not consider this, leading to infection persistence. Specifically, some authors have described how 23S rRNA gene mutations can be associated with azithromycin resistance [94]. No recent studies confirm the relevant 23S rRNA mutations to predict CT azithromycin resistance, meaning that CT azithromycin resistance is rare [85].

Notwithstanding, despite the absence of laboratory proof, it does not necessarily mean that there is no association. In detail, it is important to analyze Niekerk and colleagues’ study design and the limitations, such as the reduced sample size and the epidemiological characteristics of the participants (for example, convenience sampling causes selection bias), to better conclude if there is plausibility for this association. Regarding tetracycline resistance, reports defend its possible association with tet(M) gene mutations [95,96]. Others reported that *gyrA*, *parC*, and *ygeD* gene mutations can provide fluoroquinolone resistance [96]. In line with this, we could understand that there are some conflicting studies, reflecting a lack of fundamental studies to gather robust evidence to reach a conclusion regarding the plausibility of the association between clinically relevant gene mutations and antibiotic resistance.

Therefore, the treatment strategy must be improved, moving from a “one-size-fits-all” model to a more personalized treatment approach, according to each patient’s particular case; specifically, evaluating the presence of some bacterial mutations. Curiously, some authors have published an interesting work regarding an alternative potential treatment for bacterial STIs, such as CT and NG infections. It defends the potential role of bacteriophage therapy, a method especially efficient for gram-positive bacteria, which, unlike CT and NG (gram-negative bacteria), do not have cell membranes and a cell wall, making them an easy target for this strategy. However, despite additional research needed to prove the success of this strategy as a treatment option, there are efforts to try to overcome the challenges of this, using biological engineering techniques such as cell-penetrating peptides [97].

As for NG infections, the new window of opportunity for treating CT infections includes new drug development, specifically non-antibiotic therapy, or the drug repurposing strategy as an alternative, to “save time” [98]. Recently, some authors have published interesting findings suggesting the use of 4EpDN cyclic peptomer as a prophylactic CT treatment [99]. Also, Kazakova et al. investigated the role of C-ring oxygen and nitrogen erythrodiol derivativesin treating CT [100]. Others have targeted CT more specifically, using peptide-based inhibitors [100,101]. However, these studies need further research to conclude the promising role of such molecules and to decide if these results could be translated into clinical practice.

Investigators do their best to fight these infections. Nevertheless, despite these efforts, infection control is still hard to achieve. In line with that, maybe the critical strategy to solve these challenges would be to focus on the individuals who present some resistance to the infections. As demonstrated last year by Su et al., cGAS-STING signaling pathway played an important role in innate immunity for combating CT infections, in the female mice lower genital tract [102]. This interesting finding must be studied in humans.

In fact, vaccination would be the crowning achievement against both infections. However, such a strategy is still challenging, despite the vast efforts made during recent years and notwithstanding some ongoing trials that could bring new findings and progress [93].

## 5. Discussion of Future Directions in CT and NG Infection Control

As previously mentioned, CT and NG infections are a significant concern in developed countries because of the rising number of infected people and the consequent high costs for the economy and the patients [103]. Therefore, due to the urgency in these health problems’ control, some developed countries have implemented a screening program within the national health system to detect and treat these infections in a timely way, before irreversible injuries form [104,105,106]. Moreover, some governmental health institutions, such as the US CDC, have created guidelines regarding recommendations for the screening of several STIs, namely, CT and NG infections in distinct subsets: women, pregnant women, MSM, heterosexual men, transgender and gender-diverse persons, and people living with HIV [107]. Notwithstanding, despite the evidence and health institutions’ recommendations, these screening programs are not implemented in a generalized manner, due to the lack of funding and policy support, or the weakness of the health system [108]. Of note, another reason that could explain the non-implementation of these screening programs, as recently discussed by van Bergen and colleagues, is that it is not a linear decision, as it must consider the benefit-to-harm CT-screening ratio. They assert that even though it seems an excellent strategy for infection management, preventing new infection, and treating the infected person in a timely way, actually, it can have a “dark side”, the overuse of antibiotics that can trigger resistance [109]. This study highlights the fact that there is a critical lack of studies on CT screening and the infection itself. To effectively understand the “real world” benefits in the population, there is an urgent need for updated evidence regarding infection prevalence reduction, PID and infertility prevention, antibiotic resistance impact, and CT clearance. Fulfilling these knowledge gaps is crucial to understanding whether or not the paradigm shift from a “test-and-treat” strategy to one with new testing policies, must be considered. The authors suggest an updated Cochrane review and also updated CDC and WHO guidelines, which would be helpful in the assessment of more robust evidence and in establishing consensus recommendation strategies regarding the prevention of future CT infections [109].

The vaccine strategy would be the better way to deal with these infections, avoiding antibiotic resistance and giving immunity to all individuals, protecting them from future potential contact with the pathogens. However, CT and NG vaccines are unavailable, despite the enormous efforts made over the years [93]. It is relevant to mention that, recently it was proved that cross-protectivity was provided by the *N. meningitidis* serogroup B vaccine, MeNZB, for NG infections [110]. In addition, despite the low protectivity, the hope of effective vaccine development is increasing, due to the additional antigen mining new tools to boost this research process. Thus, the future of the vaccine strategy development would be more focused on in silico approaches, which, concomitantly with the proteomic advances, may allow the most rapid identification of new targets and the eventual development of a successful vaccine [111]. In addition, some authors assert that, while vaccines or new effective drugs are not available, it would be an excellent strategy to apply some personalized medicine in the treatment, to identify patient-specific drug susceptibility through the pathogen genotyping and phenotyping [112,113]. Therefore, while a vaccination strategy is still unavailable, this could be the key to the near-future treatment of these infections, to ultimately prescribe only the effective drugs for each infected individual, avoiding multiple drug resistance to all the available antibiotics [112].

Importantly, besides CT and NG infection control, which is also used for other STIs, primary prevention strategies should be given relevance. These types of strategies should play a more significant role, mainly at school, where educational programs should be implemented to talk to adolescents about these diseases and explain to them the preventive measures to consider, such as using condoms, to protect their health. It is important to remember that some circumstances can contribute to a higher risk of having CT or NG. In particular, individuals at higher risk of STIs, specifically HIV, can use pre-exposure prophylaxis (PrEP). The higher risk occurs because these individuals could wrongly think this is a magic bullet that allows sexually risky sexual behaviors because it eliminates the chance of HIV. In fact, it increases the risk of catching another STI, such as CT or NG, and if not mentioned and explained very carefully, they would participate in more risky sexual behavior [114].

## 6. Conclusions

Chlamydial and gonococcal infections are among the most common STIs around the world. The infection rates are the highest in individuals aged <25 years old. These infections are mostly asymptomatic, and can lead to long-term severe complications in both sexes, although the worse outcomes occur in women. As the symptoms of both infections are very similar, and the evidence shows that co-infection frequently occurs, we assert that the diagnosis technique must comprise CT and NG testing, in a dual-test strategy.

Moreover, when it comes to bacterial infections that must be treated with antibiotics, it is common for resistance and multi-resistance to these medications to arise and become additional barriers to treating CT and NG infections. In this regard, focusing on gonococcal infection is essential, due to its highest resistance rate reported by several authors.

Finally, to overcome these completely preventable health problems, all the governmental institutions must invest more in primary prevention, sharing information, and promoting STI literacy in society, and while they are doing this, also consider infection control through screening programs. In brief, it is essential to bear in mind that these health problems affect multiple layers of society, not only in the economic field but also in their social and family negative impacts. In recent years, it has been noteworthy that some countries have developed strategies to fight against CT and NG, establishing recommendation guidelines, monitoring antibiotic resistance, and making efforts in vaccine development. Nevertheless, there is still the need for more studies to fully understand the mechanisms behind this bacteria resistance and the mechanism of infection concerning infertility and, potentially, cancer development. With this study, we hope to contribute to finding the knowledge gaps regarding CT and NG infections, encouraging future investigations in this field. We have also discussed the future directions of the study of these infections, to highlight the most pertinent topics to pursue in other investigations.

## Figures and Tables

**Table 1:** Main FDA-approved CT/NG tests. Mgen—*Mycoplasma genitalium*; *T. vaginalis*—*Trichomonas vaginalis*; CLIA—clinical laboratory improvements amendment.

NAATs	Source Type	Control	Method	Target	Limitations
BD CTGCTV2 or BD^TM^ MAX^TM^ CT/GC/TV (Becton Dickinson and Company; Franklin Lakes, NJ, USA)	Vaginal, endocervical or gynecological swab; urine.	Sample processing control	PCR	CT/NG and *Trichomonas vaginalis*	Only allow genital infection diagnosis; co-infections could affect test performance [60].
Alinity m STI Assay (Abbott Molecular, Inc.; Des Plaines, IL, USA)	Vaginal, endocervical, or gynecological specimens; urine.	Independent internal and cellular controls.	RT-PCR	CT/NG, *T. vaginalis* and Mgen	False negative test could occur for Mgen, when the sample is an endocervical swab [61].
Abbott RealTime CT/NG (Abbott Molecular Inc.; Des Plaines, IL, USA)	Endocervical, vaginal, or urethral swab; urine.	Internal control	PCR	CT/NG	If asymptomatic, endocervical and male urethral swab specimens should not be used [61].
COBAS CT/NG (Roche Molecular Systems, Inc.; Rotkreuz, Switzerland)	Urine, pharyngeal, rectal, cervical, and urogenital samples.	Internal control	PCR	CT/NG	Relatively low oropharyngeal loads of NG could not be detected [62].
APTIMA Combo 2 Assay (Hologic Gen-Probe, Inc.; Marlborough, MA, USA)	Urine, vaginal, pharyngeal, rectal, and endocervical samples.	Positive and negative control	Transcription-Mediated Amplification	CT/NG	Still requires a laboratory-based platform [63].
BDProbeTec ET CT and NG Amplified DNA Assays (Becton Dickinson Microbiology Systems; Franklin Lakes, NJ, USA)	Endocervical and urethral swabs; urine.	Amplification control	Strand Displacement Amplification	CT/NG	Lower sensitivity in urine samples.
GEN-PROBE PACE 2C System for Chlamydia trachomatis and Neisseria gonorrhoeae (Gen-Probe, Inc.; Marlborough, MA, USA)	Endocervical specimens.	Two positive controls	Nucleic acid hybridization technique	CT/NG	Test limited to endocervical specimens.
Hybrid Capture II CT/GC Test (QIAGEN N.V.; Hilden, Germany)	Cervical specimens.	Internal control	Nucleic acid hybridization technique	CT/NG	Test with lower sensitivity [64].
Xpert CT/NG (Cepheid; Sunnyvale, CA, USA)	Urine, pharyngeal, rectal, vaginal and endocervical samples.	Sample processing control, sample adequacy control and probe check control.	PCR	CT/NG	It is not currently a CLIA-waived test (it must be performed in specific laboratories used to moderate- or high-complexity testing) [65].

## Data Availability

Not applicable.
